# Expert group recommendation on inhaled mucoactive drugs in pediatric respiratory diseases: an Indian perspective

**DOI:** 10.3389/fped.2023.1322360

**Published:** 2023-12-04

**Authors:** Meenu Singh, Sneha Varkki, Ilin Kinimi, Rashmi R. Das, Jagdish Prasad Goyal, Mushtaq Bhat, Rajeshwar Dayal, Pawan Kalyan, Jitender Gairolla, Indu Khosla

**Affiliations:** ^1^ Department of Pediatrics, All India Institute of Medical Sciences (AIIMS), Rishikesh, India; ^2^ Department of Pediatrics, Christian Medical College, Vellore, India; ^3^ Department of Pediatrics, Manipal Hospitals, Bengaluru, India; ^4^ Department of Pediatrics, All India Institute of Medical Sciences (AIIMS), Bhubaneswar, India; ^5^ Department of Pediatrics, All India Institute of Medical Sciences (AIIMS), Jodhpur, India; ^6^ Department of Pediatrics and Neonatology, Sher-I-Kashmir Institute of Medical Sciences, Srinagar, India; ^7^ Department of Pediatrics, Sarojini Naidu Medical College, Agra, India; ^8^ Department of Pediatrics, Dr Pinnamaneni Siddhartha Institute of Medical Sciences and Research Foundation, Chinaoutapally, India; ^9^ Department of Microbiology, All India Institute of Medical Sciences (AIIMS), Rishikesh, India; ^10^ Dr Indu’s Newborn and Pediatric Center, Mumbai, India

**Keywords:** mucoactive, inhaled, pediatric, respiratory disorders, consensus, India

## Abstract

**Background:**

Currently, there are no guidelines or consensus statements about the usage of inhaled mucoactive drugs in pediatric respiratory disease conditions from an Indian perspective.

**Objective:**

To develop a practical consensus document to help pediatricians in clinical decision-making when choosing an appropriate mucoactive drug for the management of specific respiratory disease conditions.

**Methods:**

A committee of nine experts with significant experience in pediatric respiratory disease conditions and a microbiological expert constituted the panel. An electronic search of the PubMed/MEDLINE, Cochrane Library, Scopus, and Embase databases was undertaken to identify relevant articles. Various combinations of keywords such as inhaled, nebulized, mucoactive, mucolytic, mucokinetic, expectorants, mucoregulators, mucociliary clearance, respiratory disorders, pediatric, cystic fibrosis (CF), non-CF bronchiectasis, acute wheezing, asthma, primary ciliary dyskinesia (PCD), critically ill, mechanical ventilation, tracheomalacia, tracheobronchomalacia, esophageal atresia (EA), tracheoesophageal fistula (TEF), acute bronchiolitis, sputum induction, guideline, and management were used. Twelve questions were drafted for discussion. A roundtable meeting of experts was conducted to arrive at a consensus. The level of evidence and class of recommendation were weighed and graded.

**Conclusions:**

Inhaled mucoactive drugs (hypertonic saline, dry powder mannitol, and dornase alfa) can enhance mucociliary clearance in children with CF. Experts opined that hypertonic saline could be beneficial in non-CF bronchiectasis, acute bronchiolitis, and PCD. The current state of evidence is inadequate to support the use of inhaled mucoactive drugs in asthma, acute wheezing, tracheomalacia, tracheobronchomalacia, and EA with TEF.

## 1. Introduction

**Figure 1:**
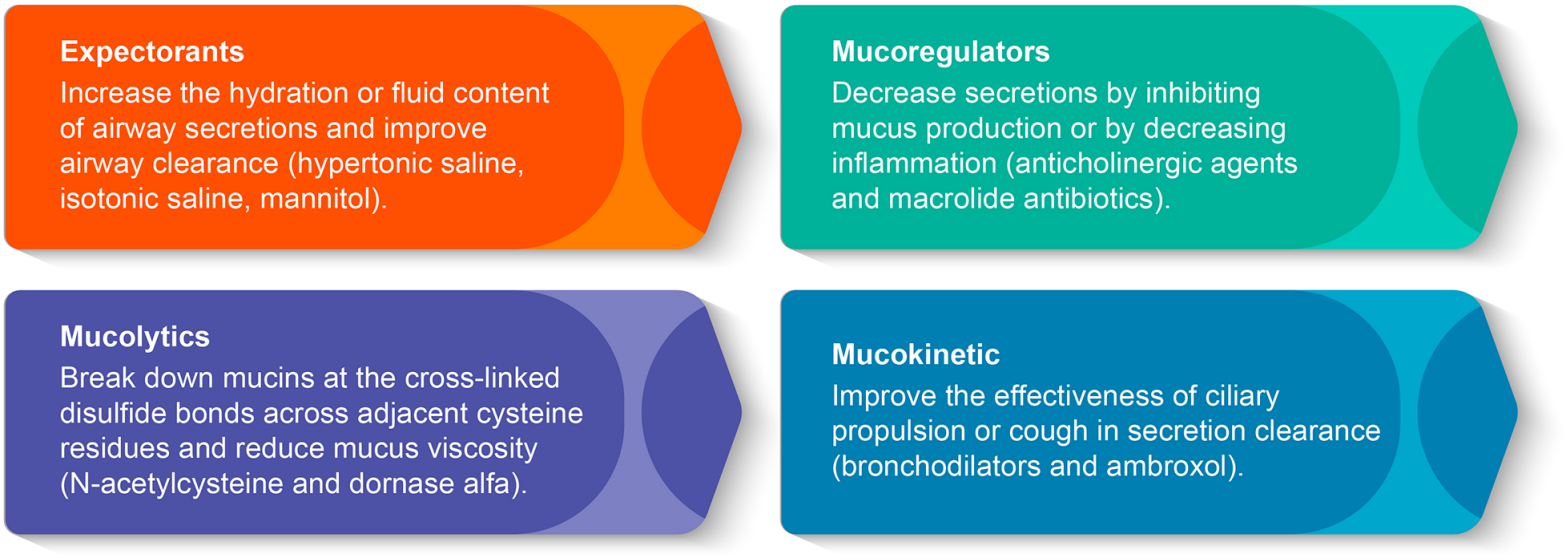
Classification of mucoactive drugs by the mechanism of action.

India has a high burden of acute and chronic respiratory diseases. Pediatric respiratory diseases place a substantial financial and human resource strain on our healthcare system every year. Several childhood disorders, such as primary ciliary dyskinesia (PCD), cystic fibrosis (CF), non-CF bronchiectasis, and severe asthma exhibit airway mucus hypersecretion (1). Mucoactive drugs have a long and well-established record of being an effective therapy for the management of respiratory diseases in which mucus hypersecretion is a clinical challenge (1, 2). Mucoactive drugs are classified as expectorants, mucoregulators, mucolytics, or mucokinetic drugs based on their potential mechanism of action (Figure 1) (1, 2). Inhaled mucoactive drugs are delivered directly to the airway and are used to improve mucus properties and reduce the mucus load in the lungs of patients suffering from muco obstructive pulmonary illness (1, 2). In this article, we have attempted to review the available literature and summarize recommendations on the role of inhaled mucoactive drugs in pediatric respiratory disease conditions from an Indian perspective.

## 2. Methodology

### 2.1. Panel selection

A panel consisting of nine experts (mean age: 53.5 years; specialty: pediatrics) with significant experience in pediatric respiratory disease conditions and a microbiological expert participated in the development of this consensus manuscript (Supplementary Table S1). Panel members were carefully selected based on their wide clinical expertise and knowledge in the field. A minimum of 10 years of clinical expertise in the field was mandatory. A moderator was identified among the panel to drive the consensus process.

### 2.2. Evidence review

An electronic search of the PubMed/MEDLINE, Cochrane Library, Scopus, and Embase databases was undertaken to identify relevant articles between January 1980 and August 2022. Various combinations of keywords such as “inhaled,” “nebulized,” “mucoactive,” “mucolytic,” “mucokinetic,” “expectorants,” “mucoregulators,” “mucociliary clearance,” “respiratory disorders,” “pediatric,” “cystic fibrosis,” “non-cystic fibrosis bronchiectasis,” “acute wheezing,” “asthma,” “primary ciliary dyskinesia,” “critically ill,” “mechanical ventilation,” “tracheomalacia,” “tracheobronchomalacia,” “esophageal atresia,” “tracheoesophageal fistula,” “acute bronchiolitis,” “sputum induction,” “guideline,” and “management” were used. Appropriate variations in search phrases and Boolean operators (AND, OR) were used. Randomized controlled trials, case reports, practice guidelines, systematic literature reviews, and meta-analyses were included. Animal studies and studies published in a language other than English were excluded. Duplicates were removed during the screening procedure. After an extensive search, 12 clinically relevant questions (Supplementary Table S2) were drafted to facilitate discussion. A virtual meeting was conducted on 24 June 2022 to finalize the questionnaire. Key articles were shortlisted and circulated among the expert panel members.

### 2.3. Consensus process

The class of recommendation (COR) and certainty of evidence (COE) were weighed and graded according to predefined scales as outlined in Table 1 (3–5). The COR was based on the grading system used by Knuuti et al., which was suitably modified and adapted to current settings (3). A roundtable meeting of experts was held on 23 August 2022 to finalize the recommendations on the role of inhaled mucoactive drugs in pediatric respiratory disease conditions. To assess the COE, we employed the Grading of Recommendations Assessment, Development, and Evaluation (GRADE) technique, as defined in the GRADE handbook (4, 5). The COE for each of the outcomes was independently evaluated by two authors. We rated the evidence from RCTs as being of high quality and downgraded it to one level for serious (or two levels for very serious) limitations based on the following considerations: design (risk of bias), consistency across studies, directness of evidence, precision of estimates, and presence of publication bias. After the group discussion, clinical consensus statements were formulated based on the opinions and agreement of the majority. During group discussions, all panelists were encouraged to participate actively. The differences in opinions were discussed and resolved. Certain recommendations are based on the collective clinical judgment from real-world practice and no grading of recommendations has been applied for the same. A draft of the clinical consensus statements and recommendations was circulated among the expert panel for review. After the second meeting, the experts discussed updating any new findings (if any). A second round of basic literature searches was conducted in PubMed/MEDLINE, Cochrane Library, Scopus, and Embase databases in June 2023 to check for any new updates/findings. The final draft of the clinical consensus statements and recommendations was circulated among the expert panel for final review and approval in the first week of July 2023.

**Table 1:** Definitions: (A) class of recommendation; and (B) certainty of evidence.

1. Class of recommendation (COR)		
Class I	“Evidence and/or general agreement that a given treatment or procedure is beneficial, useful, or effective”	“Agreement” (“Recommended” or “indicated”)
Class II	“Conflicting evidence and/or a divergence of opinion about the usefulness/efficacy of the given treatment or procedure”	“Conditional agreement” (“May be considered”)
Class III	“Evidence or general agreement that the given treatment or procedure is not useful/effective, and in some cases may be harmful”	“Disagreement” (“Not recommended”)
2. Certainty of evidence (COE)		
High	“Further research is very unlikely to change our confidence in the estimate of the effect”	
Moderate	“Further research is likely to have an important impact on our confidence in the estimate of the effect and may change the estimate”	
Low	“Further research is very likely to have an important impact on our confidence in the estimate of the effect and is likely to change the estimate”	
Very low	“Any estimate of effect is very uncertain”	

Adapted from: Knuuti et al. (3), GRADE handbook for grading quality of evidence and strength of recommendations (4) and Atkins et al. (5).

## 3. Results

### 3.1. Cystic fibrosis

Cystic fibrosis is a genetic illness caused by a gene defect on chromosome 7 that encodes for CF transmembrane conductance regulator (CFTR) protein (6). In patients with CF, mucociliary clearance is impaired. Evidence (Table 2) showed that inhaled hypertonic saline (HS; an expectorant) enhanced mucociliary clearance (15), improved lung clearance index (8, 16), and reduced pulmonary exacerbations (17) in children with CF as compared with isotonic saline. Salbutamol followed by inhaled 3% HS positively affected structural lung changes relative to 0.9% saline (9). Higher HS strengths (5%, 6%, or 7%) may have the same or better effect. However, more research is needed in a developing country setting like India. In children with CF, the use of inhaled mannitol, a hyperosmotic mucoactive drug, also resulted in an improvement in lung function [forced expiratory volume in 1 s (FEV1)] (12, 18, 19). Clinical evidence supports the use of recombinant human DNAase I (rhDNase; dornase alfa), a mucolytic drug as it improved lung function (FEV1) and reduced pulmonary exacerbations in children with CF (20, 21). N-acetylcysteine (NAC), a mucolytic drug, causes cleavage of disulfide bonds to two sulfhydryl groups, resulting in thinning of the mucus (1). No beneficial effect of NAC on lung function has been reported in children with CF (14). Heparin inhalation showed no significant effect on sputum clearance, FEV1, or sputum inflammatory markers in adults with CF (22). Currently, there is no evidence of the role of inhaled heparin in children with CF.

**Table 2:** Clinical studies of inhaled mucoactive drugs in CF.

Author and year	Study design and study groups	Key findings	Quality/certainty of evidence (per GRADE criteria)
HS and NS			
Wark et al. (7)	Systematic review	CF with stable lung disease (246 participants from four trials): 4 HS (3%–7% vs. placebo) improved FEV_1_ (MD: 3.3%) at 4 weeks. 5 HS improved LCI by 0.6 units (MD) at 48 weeks vs. NS (two trials; 192 participants). 6 The study reported uncertainty around the impact of HS and differences in mucociliary clearance, pulmonary exacerbations, or AEs vs. placebo.	Very low COE. More data are needed.
		CF with acute exacerbation (130 participants from one trial): • No significant difference in FEV_1_ between HS and NS/placebo (MD: 5.1%) in FEV_1_ at 4 weeks • No serious AEs were reported	Very low COE More data are needed.
Ratjen et al. (8)	• Multicenter, double-blind RCT • Studied the impact of twice-daily inhaled 7% HS on LCI_2–5_ in preschool children with CF vs. NS.	150 preschool children (3–6 years of age) with early CF lung disease. • HS led to a significant improvement in LCI_2–5_ by 0.63 units in 48 weeks. • None of the serious AEs were treatment-related.	Low COE More data are needed.
Tiddens et al. (9)	6 Multicenter, double-blind RCT. 7 Evaluated the effect of inhaled 7% HS on chest CT in preschool children with CF. 8 Salbutamol followed by either 7% HS or NS twice daily for 48 weeks.	116 preschool children (3–6 years of age) with early CF lung disease. • HS led to a significant positive effect on structural lung disease based on chest CT imaging.	Low COE More data are needed.
HS (3%–7%) vs. rhDNase			
Wark et al. (7)	Systematic review	CF with stable lung disease (61 participants from two trials): 6 No significant difference in FEV_1_ between HS and rhDNase (MD: 1.6%) in FEV_1_ at 3 weeks. • rhDNase led to a greater increase in FEV_1_ (MD: 8%) at 3 months. • Other outcomes were not reported.	Very low COE More data are needed.
			Low COE More data are needed.
HS (3%–7%) vs. mannitol			
Wark et al. (7)	Systematic review	CF with stable lung disease (12 participants from one trial): • Lung function outcomes were not reported at different time points. • No difference in sputum clearance between the HS and mannitol study arms. • Mannitol was reported to be more irritating compared with HS.	Very low COE More data are needed.
HS 3% vs. 7%			
Wark et al. (7)	Systematic review	CF with stable lung disease (30 participants from one trial): • The study reported uncertainty about whether there was an improvement in FEV_1_% predicted after treatment with 7% HS vs. 3% HS.	Very low COE More data are needed.
Timing of HS administration			
Elkins et al. (10)	Systematic review	CF with stable lung disease (77 participants from three trials): • Before or during ACT may maximize perceived efficacy and satisfaction. • The study highlighted that HS inhalation before or during ACT may maximize perceived efficacy and satisfaction. • Twice-daily inhalations have been shown to have long-term efficacy. Until information comparing various regimens is available, the time of day at which it is inhaled may be determined by convenience or tolerability if only one dose per day is tolerated.	Low COE More data are needed.
Inhaled dry powder mannitol vs. nonrespirable mannitol or rhDNase or HS			
Nevitt et al. (11)	Systematic review	CF with stable lung disease (784 participants from six studies): • The study found no difference in QoL between the mannitol and control groups or mannitol administered with or without additional rhDNase. • The review highlighted improvements in some measures of lung function (FEV_1_ [ml], FEV_1_% predicted, FVC [ml], FEF_25–75_ ml/s) with mannitol (over 6 months) vs. control. • The occurrence of side effects (cough and bronchospasm) was not significantly different between the mannitol and control arms. • No studies compared mannitol vs. HS.	Low to very low COE. More data are needed. Moderate COE Low COE More data are needed.
Sadr et al. (12)	• RCT • Assessed the efficacy of soluble mannitol (150 mg/ml; twice a day) in NS vs. inhaled 5% HS (four times a day) for 2 weeks in children (≥5 years of age) with CF. • Note: Both groups received inhaled salbutamol pretreatment.	CF with stable lung disease (30 participants from one trial): • Inhaled soluble mannitol significantly improved pulmonary function (FEV_1_) that was not seen with HS (5%) at 2 weeks of treatment.	Very-low COE More data are needed.
rhDNase vs. placebo, no treatment, or HS			
Yang et al. (13)	Systematic review	Dornase alfa compared with placebo or no treatment (1,708 participants from eight trials): • Dornase alfa improved lung function within 1 month. • Fewer pulmonary exacerbations or flare-ups of lung inflammation on long-term use.	Moderate to high COE
		Daily vs. alternate day (43 participants from one trial): • No difference between daily vs. alternate day treatment schedules of rhDNase on lung function, QoL, or pulmonary exacerbations.	Low COE More data are needed.
		Compared with HS (43 participants from one trial): • Greater improvement of lung function with rhDNase vs. HS.	Low COE More data are needed.
NAC (inhaled or oral) vs. placebo			
Duijvestijn et al. (14)	Systematic review	Nebulized NAC (28 participants from three trials): • No beneficial effect on lung function.	Very low COE More data are needed.
		Oral NAC (181 participants from six trials): • The tendency towards a beneficial effect on lung function of oral NAC therapy on FEV_1_, it was small and of doubtful clinical relevance.	Very low COE More data are needed.

ACT, airway clearance technique; AE, adverse event; CF, cystic fibrosis; COE, certainty of evidence; CT, computed tomography; FEF_25–75_, forced expiratory flow at 25%–75% of FVC; FEV_1_, forced expiratory volume in 1 s; FVC, forced vital capacity; GRADE, grading of recommendations assessment, development and evaluation; HS, hypertonic saline; LCI, lung clearance index; MD, mean difference; NAC, N-acetylcysteine; NS, normal saline; QoL, quality of life; RCT, randomized controlled trial; rhDNase, recombinant human deoxyribonuclease I.

#### 3.1.1. Expert opinions/consensus recommendations

Children with CF would benefit from initiation of HS inhalation (6% or 7%; twice or thrice daily) from the time of diagnosis (COR: I, agreement, very low CoE).

The experts concurred that HS inhalation (6% or 7%; twice or thrice daily) as an adjunct to the airway clearance technique (ACT) can enhance mucociliary clearance and reduce pulmonary exacerbations in children with CF based on real-world practice (COR: II, conditional recommendation, very low CoE). As local side-effects are common after a higher strength of HS (6% and 7%) that may affect tolerability, more research on 3% HS is needed.

Inhaled dry powder mannitol (400 mg; twice daily) is useful for clearing retained airway secretions in children with CF (COR: II, conditional recommendation, very low to low CoE). However, a mannitol dry powder inhaler is currently unavailable in India for managing mucus hypersecretion in children with CF. The experts suggested that tolerability testing is needed before treatment.

Dornase alfa (rhDNAse; 2.5 mg; once or twice daily) in nebulized form is useful in reducing the risk of exacerbations of respiratory symptoms requiring parenteral antibiotics in children with CF (COR: I, agreement, moderate to high COE). However, rhDNase is currently unavailable in India and the cost associated with therapy is high for managing mucus hypersecretion in children with CF.

The use of NAC is not beneficial in children with CF. However, more data are needed (COR: III, disagreement, very low COE).

### 3.2. Non-CF bronchiectasis

Bronchiectasis is a chronic respiratory disease associated with wet cough in children and recurrent infective exacerbations impacting the quality of life (QoL) (23, 24). Treatment for non-CF bronchiectasis consists of management of nutrition, airway clearance, and antibiotics for exacerbations (23, 24). Tarrant et al. systematically reviewed the effects of mucoactive drugs in chronic non-CF bronchiectasis. Both HS (6% or 7%) and normal saline (0.9% sodium chloride; NS) improved FEV1, forced vital capacity (FVC), and forced expiratory flow25%–75% (FEF25%–75%) in bronchiectasis after one dose and after 3–12 months (25). Mannitol failed to improve spirometry in bronchiectasis. On the contrary, rhDNase caused significant reductions in FEV1 and FVC, but increased exacerbation rate, and reduced spirometry (25). Another review of inhaled HS in bronchiectasis found that it improved expectoration, reduced sputum viscosity, improved lung function, and reduced the frequency of exacerbations (23). The British Thoracic Society guidelines for the management of non-CF bronchiectasis in adults mention that inhaled HS may be used as an adjunct to physiotherapy (26). The use of rhDNase is not advised as it worsens lung function due to an increase in exacerbation frequency. In addition, it states that there is no definitive clinical evidence to confirm its use in children or adults with bronchiectasis (26). Anuradha et al. highlighted that inhaled salbutamol (200 µg) followed by 3% HS nebulization twice daily for 8 weeks before chest physiotherapy significantly improved FEV1 in children (*N* = 26; 5–15 years of age) with non-CF bronchiectasis (27). In addition, improvement in FVC and reduction in the frequency of exacerbation were significant compared with conventional ACT (*N* = 26; inhaled salbutamol before chest physiotherapy) (27). Table 3 lists clinical studies of inhaled mucoactive drugs in chronic non-CF bronchiectasis.

**Table 3:** Clinical studies of inhaled mucoactive drugs in chronic non-CF bronchiectasis.

Author and year	Study design	Key findings	Quality/certainty of evidence (per GRADE criteria)
HS vs. NS			
Tarrant et al. (25)	Systematic review	Chronic non-CF bronchiectasis (92 adult participants from three trials): • One trial of a factorial design with patients randomized to receive: (i) active cycle breathing; (ii) nebulized terbutaline then active cycle breathing; (iii) nebulized terbutaline, NS then active cycle breathing; and (iv) nebulized terbutaline, HS [7%] then active cycle breathing). In terms of lung function improvement and sputum clearance at 3 months, this trial discovered that HS was better than other therapies (28) • The other trial compared HS [7%] vs. NS in a cross-over design. Improvement in lung function, QoL, and healthcare utilization was better in the HS group (29) • The third trial compared HS [6%] vs. NS. There was no difference in the number of exacerbations, QoL, sputum colonization, and respiratory function over 12 months (30)	Low COE (based on the pooled data from these trials). Data needed in the pediatric population.
Mannitol vs. placebo			
Tarrant et al. (25)	Systematic review	Chronic non-CF bronchiectasis [804 pediatric (adolescent) and adult participants from two trials]: • The two trials found that mannitol was no better than the control in terms of improvement of QoL. AEs were similar between the two groups (31, 32). However, in one trial the time to first pulmonary exacerbation was increased on mannitol (32).	Low COE Data exclusively on the pediatric population are needed.
rhDNase vs. placebo			
Tarrant et al. (25)	Systematic review	Chronic non-CF bronchiectasis (409 adult participants from two trials): • One (60 patients) trial administered rhDNase for 2 weeks (33) and the other (349 patients) for 24 weeks (34). A detrimental effect (decline in lung function and increase in exacerbation rate) was reported (34).	Moderate COE Data needed in the pediatric population.
NAC			
No trials were identified			

AE, adverse event; CF, cystic fibrosis; COE, certainty of evidence; GRADE, grading of recommendations assessment, development and evaluation; HS, hypertonic saline; NS, normal saline; NAC, N-acetylcysteine; QoL, quality of life; rhDNase, recombinant human deoxyribonuclease I.

#### 3.2.1. Expert opinions/consensus recommendations

Evidence on the efficacy and safety of inhaled HS in adults with non-CF bronchiectasis is available, and it is beneficial (low CoE). More research into the pediatric population is required. Based on the data available for adults, the experts proposed that the inhaled HS before chest physiotherapy can be tried in children with non-CF bronchiectasis until more data are available.

The HS inhalation is associated with the risk of bronchospasm. Based on real-world experience similar to that of children with CF, the experts suggested that inhaled salbutamol followed by HS before chest physiotherapy and postural drainage can be helpful in children with non-CF bronchiectasis. Multicenter RCTs are required to evaluate the efficacy and safety of inhaled HS in children with non-CF bronchiectasis.

Evidence on the efficacy and safety of inhaled mannitol in adolescents and adults with non-CF bronchiectasis is available (low CoE). However, inhaled mannitol may not be readily available in India, and data on children are required.

There are no pediatric studies that assessed the efficacy and safety of rhDNase in non-CF bronchiectasis. The data in adults show that it worsens lung function (moderate CoE). Thus, the experts agreed that, currently rhDNase should not be used in children with non-CF bronchiectasis.

### 3.3. Acute wheezing

Ater et al. studied the effectiveness of 5% HS on acute wheezing (Table 4A) in children after salbutamol inhalation relative to NS (35). Inhaled HS substantially shortened the stay and admission rate (35). In children with acute viral wheeze, HS/salbutamol significantly reduced hospital stay and oxygen therapy time, and improved asthma clinical severity score quicker than NS/salbutamol (36).

**Table 4:** Clinical studies of inhaled mucoactive drugs in under-five wheezing (A) and asthma (B).

Author and year	Study design and study groups	Key findings	Quality/certainty of evidence (per GRADE criteria)
HS vs. NS			
Under-five wheezing Ater et al. (35)	• RCT **5** Compared the efficacy of 5% HS (twice every 20 min in the ED and four times a day later if hospitalized) on wheeze in children after salbutamol inhalation to NS.	Under-five wheezing (acute, moderate to severe wheezing) (41 children): • Shorter hospital stays with HS (2 days) vs. NS (3 days). Decrease in admission rate: 62% (HS) vs. 92% (NS).	Low COE More data are needed.
Under-five wheezing Kanjanapradap et al. (36)	• RCT 6 Evaluated the effectiveness of inhaled salbutamol in 3% HS (every 4–6 h until discharge) relative to NS/salbutamol in children with wheezing.	Under-five wheezing (acute, moderate to severe wheezing) (47 children): • Shorter hospital stays with HS (2 days) vs. NS (3 days). • Decreased duration of oxygen therapy with HS (1½ days) vs. NS (3 days). • Significant improvement in asthma severity score, respiratory rates, and oxygen saturation at 12 h in the HS group.	
Asthma Teper et al. (37)	• RCT • Studied the bronchodilator response to salbutamol when inhaled with 3% HS vs. NS in asthmatic children with mild or moderate bronchial obstruction.	Stable asthma (50 children): • 6 (24%) patients in the NS group had a decrease in FEV_1_ at 30 min compared with those at baseline. • Improvement in FEV_1_ in the HS group at 30 min (Note: none of the patients had a decrease in FEV_1_ at 30 min).	

COE, certainty of evidence; ED, emergency department; FEV_1_, forced expiratory volume in 1 s; GRADE, grading of recommendations assessment, development and evaluation; GRADE, grading of recommendations assessment, development and evaluation; HS, hypertonic saline; NS, normal saline; RCT, randomized controlled trial.

#### 3.3.1. Expert opinions

HS alone has never been used to treat children with acute wheezing as it can provoke bronchospasm.

HS (5% and 3%), when used along with salbutamol, has been shown in two studies by Ater D et al. (35) and Kanjanapradap T et al. (36) to have a positive effect (shorter length of hospital stay) in preschool wheeze relative to NS. However, the experts unanimously agreed that the current state of evidence is inadequate to recommend the routine use of HS in clinical practice in children with acute wheezing. (COR: III, disagreement, low CoE).

### 3.4. Asthma

Asthma is an inflammatory chronic airway disease characterized by bronchial hyperresponsiveness and airflow obstruction. Wheezing, mucus hypersecretion, and mucus plugging are reported in patients with asthma, especially during exacerbations (37). Short-acting beta2-agonist bronchodilators, such as salbutamol and systemic corticosteroids, are usually advised for asthma exacerbations (37). It has also been seen that salbutamol produced a greater bronchodilator response (FEV1 and maximum mid-expiratory flow) when inhaled with 3% HS vs. NS in asthmatic children with mild or moderate bronchial obstruction (Table 4B) (37).

#### 3.4.1. Expert opinions/consensus recommendations

HS alone has never been used to treat children with asthma as it can provoke bronchospasm.

The current state of evidence is inadequate to recommend the routine use of HS in clinical practice for asthma. There is a need for well-designed multicenter RCTs to assess the role of HS in children with asthma. (COR: III, disagreement, very low CoE).

### 3.5. Primary ciliary dyskinesia

Primary ciliary dyskinesia is a rare disorder characterized by motile ciliary dysfunction. This leads to an array of clinical manifestations, including neonatal respiratory distress (in term infants), persistent wet cough, rhinitis without remission, chronic sinusitis (in childhood), and bronchiectasis (in adolescence) (38). Currently, there is a lack of RCTs that have assessed the effectiveness of mucoactive drugs in children with PCD. Few case studies have highlighted the use of inhaled HS and rhDNase in the management of PCD in children (39, 40). The European Respiratory Society (ERS) consensus statement suggests: (i) inhaled NS or HS to increase mucus clearance (low-quality evidence, weak recommendation); or (ii) inhaled rhDNase in selected patients with PCD (low-quality evidence, weak recommendation) (38). Pediatric PCD patients require mucus hypersecretion management when they develop bronchiectasis, also known as non-CF bronchiectasis. Table 5 lists clinical studies of inhaled mucoactive drugs in the management of PCD.

**Table 5:** Clinical studies of inhaled mucoactive drugs in PCD.

Author and year	Study design and study groups	Key findings	Quality/certainty of evidence (per GRADE criteria)
HS vs. NS			
Paff et al. (41)	• RCT • Patients received twice-daily inhalations of HS (7%) or NS for 12 weeks.	PCD (44 adult participants): • In this cross-over design, though the QoL improved more after HS inhalations than after NS inhalations, the difference was not statistically significant. • AEs were more common after HS but were mild (throat irritation, cough, and chest tightness).	Very low COE. More data are required in the pediatric population.
HS			
Kaspy et al. (42)	Retrospective study	PCD (34 infants): • Early initiation of HS nebulization during the neonatal period in PCD-diagnosed cases led to decreased hospitalization, improved nasal hygiene, and other outcomes.	Very low COE. More data are needed on infants and children.
NAC vs. placebo			
Stafanger et al. (43)	RCT	PCD (13 participants): • In this cross-over design, NAC, either 200 mg × 3 daily (patients weighing <30 kg) or 400 mg × 2 daily (>30 kg) for two 3-months periods did not find any benefit.	Very low COE. More data are needed on the pediatric population.
Dornase alfa			
No trials were identified			

AE, adverse event; COE, certainty of evidence; GRADE, grading of recommendations assessment, development and evaluation; HS, hypertonic saline; NAC, N-acetylcysteine; NS, normal saline; PCD, primary ciliary dyskinesia; QoL, quality of life; RCT, randomized controlled trial.

#### 3.5.1. Expert opinions/consensus recommendations

There is a lack of RCTs that have assessed the effectiveness of inhaled HS in children with PCD. Adult studies of inhaled HS or NS on non-CF bronchiectasis show beneficial effects. Thus, the evidence on adults can be extrapolated to pediatrics. In line with the ERS consensus statement, the experts agreed that the use of inhaled NS or HS should possibly be considered to increase mucus clearance in patients with PCD (COR: II, conditional agreement) (38).

The use of NAC is not recommended in children with PCD (COR: III, disagreement, very low CoE).

### 3.6. Critically Ill on mechanical ventilator support

Ventilator-associated pneumonia (VAP) is a serious complication related to mechanical ventilation in the neonatal period in pediatric intensive care units. Ezzeldin et al. found a significant reduction in the incidence density of VAP (Table 6) with a 3% HS group as an adjunct to VAP prevention protocol in intubated and mechanically ventilated premature infants (46).. In mechanically ventilated children after cardiac surgery, inhaled rhDNase resulted in a reduction in the duration of ventilator support by approximately 1 day and lowered the incidence of atelectasis vs. NS (47).

**Table 6:** Clinical studies of inhaled mucoactive drugs in children on mechanical ventilation.

Author and year	Study design and study groups	Key findings	Quality/certainty of evidence (per GRADE criteria)
Mucoactive agents			
Anand et al. (44)	Systematic review	Patients on mechanical ventilation (1,712 participants from three trials): • Four drugs used: NAC, HS (3%), heparin, and ambroxol. • The study did not support the use of mucoactive agents in critically ill patients with acute respiratory failure.	Low COE More data are needed.
HS vs. NS/placebo			
Shein et al. (45)	1. RCT 2. Children who had been intubated (for less than 12 h) and had an expected duration of mechanical ventilation (>48 additional hours) were given HS (3%) or NS 4 times/day	Children on mechanical ventilation (18 participants): • Nebulized HS did not improve outcomes, including the duration of mechanical ventilation. • Wheezing after HS treatment was rare.	Very low COR. More data are needed.
Ezzeldin et al. (46)	• RCT • Studied the effectiveness of 3% HS (twice daily; as an adjunct to VAP prevention protocol) to reduce the incidence of VAP in intubated and mechanically ventilated premature infants.	Preterm infants on mechanical ventilation (100 participants): • Nebulized HS may help preserve lung clearance mechanisms and reduce VAP in premature infants.	Very low COE. More data are needed.
rhDNase vs. placebo			
Riethmueller et al. (47)	• RCT 1. Studied the impact of rhDNase during postoperative ventilation on the duration of ventilator support and incidence of atelectasis vs. NS.	Infants on mechanical ventilation (100 participants): • Reduced atelectasis, reduced time on mechanical ventilation, and shorter ICU stay with rhDNase.	Very low COE More data are needed on children.
Youness et al. (48)	• RCT • Patients were randomized into three study groups: (i) rhDNase; (ii) HS (7%); and (iii) NS every 12 hours	Adults on mechanical ventilation (33 participants in a three-arm trial): • HS was no more effective than NS in this study population. • In mechanically ventilated patients with newly developed atelectasis who received twice-daily rhDNase nebulization, there was no appreciable improvement in the chest x-ray atelectasis score. HS was no more effective than NS in this study population.	Very low COE More data are needed on children.
Zitter et al. (49)	• RCT • Patients received rhDNase or placebo twice daily until extubation, death, transfer, or 30 days	Adults on mechanical ventilation (30 participants): • Over the first 5 days of treatment, rhDNase did not improve the appearance of atelectasis on chest radiographs or the overall chest x-ray score in mechanically ventilated patients.	Very low COE More data are needed on children.
NAC vs. NS			
Masoompour et al. (50)	• RCT • Patients were randomized to receive NAC or NS thrice a day for 1 day.	Adults on mechanical ventilation (40 participants): • NAC was not effective more than NS at lowering the density of mucous plugs in mechanically ventilated patients.	Very low COE. More data are needed on the pediatric population.

COE, certainty of evidence; GRADE, grading of recommendations assessment, development and evaluation; HS, hypertonic saline; NAC, N-acetylcysteine; NS, normal saline; RCT, randomized controlled trial; rhDNase, recombinant human deoxyribonuclease I; VAP, ventilator-associated pneumonia.

#### 3.6.1. Expert opinions/consensus recommendations

Inhaled HS (3%) has been shown to reduce the incidence density of VAP in intubated and mechanically ventilated premature infants. However, further research is needed in children (COR: III, disagreement, very low CoE).

Dornase alfa has been shown to reduce the length of stay and duration of ventilation in intubated and mechanically ventilated infants. However, further research is needed in children (COR: III, disagreement, very low CoE). It is also not available in India.

In adults, NAC has not been shown to be more effective than NS (COR: III, disagreement, very low CoE). Further data are needed on the pediatric population.

### 3.7. Tracheomalacia and tracheobronchomalacia

Tracheomalacia and tracheobronchomalacia have been increasingly recognized in children in recent years. Depending on the site and severity of the lesion, clinical presentation includes early onset stridor or fixed wheeze, recurrent infections, and cough (51). Isotonic saline or HS can aid in mucus clearance (52). Boogaard et al. found that in children with airway Tracheobronchomalacia and lower respiratory tract infections (Table 7A), the use of inhaled rhDNase did not enhance recovery from respiratory symptoms markedly cough, dyspnea, and difficulty in sputum expectoration (56).

**Table 7:** Clinical studies of inhaled mucoactive drugs in children with tracheobronchomalacia and EA with TEF.

Author and year	Study design	Key findings	Quality/CoE (per GRADE criteria)
14 Tracheobronchomalacia (rhDNase vs. placebo)			
Goyal et al. (53)	Systematic review	Children with airway tracheobronchomalacia and respiratory infection (40 from one trial): • Inhaled rhDNase did not enhance recovery from respiratory symptoms (cough, dyspnea, and difficulty in sputum expectoration).	Very low COE More data are needed.
15 EA with TEF (10% nebulized NAC vs. NS)			
Singh et al. (54)	Non-RCT (Intervention study)	Children (30 participants): • Nebulized NAC led to decreased consistency of secretions, earlier discharge, and a higher survival rate vs. the control group.	Very low COE More data are needed.
Pandey et al. (55)	Observational study	Children (7 participants): • Nebulized NAC given in both preoperative and postoperative periods led to earlier surgery and a smooth postoperative course.	

COE, certainty of evidence; EA with TEF, esophageal atresia with tracheoesophageal fistula; GRADE, grading of recommendations assessment, development and evaluation; NAC, N-acetylcysteine; NS, normal saline; RCT, randomized controlled trial; rhDNase, recombinant human deoxyribonuclease I.

#### 3.7.1. Expert opinion

There is limited evidence regarding the role of inhaled mucoactive drugs in patients with tracheomalacia and tracheobronchomalacia. Further research evaluating the efficacy and safety of inhaled mucoactive drugs in tracheomalacia and tracheobronchomalacia is needed in pediatric patients. (COR: III, disagreement, very low CoE).

### 3.8. Esophageal atresia with tracheoesophageal fistula

Congenital esophageal atresia (EA with tracheoesophageal fistula (TEF is a rare condition that occurs in 1 per 3,000 live births. Recurrent pneumonia, wheezing, and persistent cough are noted in these children (57). Inhaled NAC (Table 7B) has shown promising results in liquefying the airway secretions in EA with TEF and patients were discharged earlier than when treated with NS (54).

#### 3.8.1. Expert opinions/consensus recommendations

There is limited evidence regarding the role of inhaled NAC in patients with EA with TEF. More prospective RCTs are required to make strong recommendations. (COR: III, disagreement, very low CoE).

### 3.9. Acute bronchiolitis

Acute bronchiolitis is a common cause of hospitalization and morbidity in infancy (58). The mainstay of therapy for acute bronchiolitis includes airway support, gentle nasal suctioning, fluid administration, and adequate nutrition (58, 59). Evidence (Table 8) suggests that inhaled HS shortened the length of hospital stay and improved the clinical severity score (in the first 3 days) in children with acute bronchiolitis (58, 63–65). Furthermore, treatment with inhaled HS may also substantially reduce the risk of hospitalization among outpatients and emergency department patients (58). Only one study assessed the effectiveness of inhaled NAC solution in children with acute bronchiolitis. In children with acute viral bronchiolitis, inhaled NAC in NS displayed an improvement in clinical severity score and resulted in early discharge from the hospital in children relative to salbutamol (62).

**Table 8:** Clinical studies of inhaled mucoactive drugs in children with acute bronchiolitis.

Author and year	Study design and study groups	Key findings	Quality/certainty of evidence (per GRADE criteria)
HS vs. NS/standard care			
Zhang et al. (60)	Systematic review	Infants (5,205 from 34 trials): • Modest reduction in length of stay in hospitalized infants and a slight improvement in clinical severity score. • Reduction in the risk of hospitalization amongst patients with outpatients and ED. • Minor and spontaneously resolved AEs, particularly when administered in conjunction with a bronchodilator.	Low COE. More data are needed.
Nebulized rhDNase alone or in combination vs. placebo			
Enriquez et al. (61)	Systematic review	Infants (333 from three trials): • Treatment did not reduce hospitalization time or enhance clinical outcomes. It could be useful in severe bronchiolitis complicated by atelectasis.	Very low COE. More data are needed.
NAC			
Naz et al. (62)	1. Prospective RCT 2. Compared the efficacy of inhaled NAC (20 mg) in NS vs. salbutamol (2.5 mg) in NS as inhaled aerosol thrice daily for 5 days.	1. As compared with salbutamol, inhaled NAC displayed improvement in clinical severity score and resulted in early discharge from the hospital in children (*N* = 100; 2–24 months of age). 2. No AEs were observed.	Very low COE. More data are needed.

AE, adverse event; COE, certainty of evidence; ED, emergency department; GRADE, grading of recommendations assessment, development and evaluation; HS, hypertonic saline; NS, normal saline; RCT, randomized controlled trial; NAC, N-acetylcysteine; rhDNase, recombinant human deoxyribonuclease I.

#### 3.9.1. Expert opinions/consensus recommendations

Inhaled HS therapy offers benefits in terms of reduced rate of hospitalization and readmission rates in infants and children with acute bronchiolitis. The experts suggested that inhaled salbutamol followed by HS (3%; every 6–8 h until discharge) may be considered in children with acute bronchiolitis (COR: II, conditional agreement, low CoE). The experts suggested that in certain phenotypes of bronchiolitis (history of atopy or wheezing), salbutamol may be considered.

Inhaled NAC is not studied well in acute bronchiolitis and is not commonly used in children with acute bronchiolitis. Multicenter RCTs are required to evaluate the efficacy and safety of inhaled NAC in children with acute bronchiolitis (very low CoE).

### 3.10. Sputum induction

Suri R et al. assessed the effectiveness (Table 9) of sputum induction (SI) with 7% inhaled HS on airway inflammation in children with CF (67). Sputum induction was found to be safe with no evidence of a proinflammatory effect. Furthermore, SI helped in the identification of organisms in culture-negative symptomatic children, circumventing the need for bronchoscopy (67). Ferreira et al. found that SI capacity was significantly increased in children with CF after 7% HS inhalation (68). Pathogen yield from SI was shown to be superior to cough swabs, and the technique can be used as a substitute for bronchoalveolar lavage in children with CF (69). Ultrasonic nebulizers are more successful in inducing sputum relative to jet nebulizers, and pretreatment with salbutamol can inhibit bronchoconstriction induced by HS inhalation (70). The usefulness of SI has also been studied in adult patients with pulmonary tuberculosis with 7% HS for improving bacteriological yield (71). The use of induced sputum samples was more sensitive than gastric washing specimens for the diagnosis of tuberculosis in patients who could not expectorate spontaneously (72).

**Table 9:** Clinical studies of inhaled mucoactive drugs in SI.

Author and year	Study design and study groups	Key findings	Quality/certainty of evidence (per GRADE criteria)
HS for sputum induction in asthma			
Gibson et al. (66)	Review Children >6 years with asthma (over 500 children from various studies): • Stable asthma (*n* = 308), acute asthma (*n* = 18), and healthy control subjects (*n* = 185). • In most studies, *β*2-agonist was used as a pretreatment.	• There fall in lung function (>10% of baseline) seen in only 6% of cases quickly reversed with *β*2-agonists.	Very low COE More data are needed.
HS for sputum induction in CF			
Suri et al. (67)	3 Crossover trial 1 Studied the efficacy and safety of SI with 7% inhaled HS in children with CF. 2 Sputum induction was performed by inhalation of 7% HS using a nebulizer compressor system for 12 min. Patients on regular bronchodilator therapy (terbutaline sulfate or salbutamol) were given inhaled bronchodilator (for 10 min) before HS.	Children (48 from 1 study): • Sputum induction was safe, with no proinflammatory effect	Very low COE. More data are needed.
Ferreira et al. (68)	1 Cross-sectional study 3 Studied the efficacy and safety of SI with 7% inhaled HS in children with CF. After salbutamol inhalation, 7% HS was administered via a face mask through a noninvasive oxygen delivery method at 3 L/min for SI.	Children (64 from 1 study): Inhalation of 7% HS increased the sputum production and detection of pathogens.	
Ronchetti et al. (69)	Intervention study (internally controlled) 2 Studied the effectiveness of SI (with 7% inhaled HS) as a diagnostic intervention for pathogen detection in children with CF. 3 Inhaled 7% HS was administered through a disposable oxygen-driven jet nebulizer at 5 L/min for 15 min.	Children (124 from one study): • SI is superior for pathogen detection (vs. cough swabs), effective in sampling lower airways, and a credible surrogate for bronchoalveolar lavage.	

CF, cystic fibrosis; GRADE, grading of recommendations assessment, development and evaluation; HS, hypertonic saline; SI, sputum induction.

#### 3.10.1. Expert opinion/consensus recommendations

Nebulization with HS (7%) may be considered to facilitate sputum expectoration even in patients who usually do not expectorate (COR: II; conditional agreement, very low CoE). This method has been applied in patients with CF to enhance mucus clearance, for the identification of infectious agents, and for cytological examination in inflammatory airway disorders. It may avoid invasive interventions, such as bronchoscopy, to obtain better samples for pathogen detection (69).

A comparison of the efficacy and safety data of various strengths of HS for SI is required.

## 4. Discussion

Currently, there are no country-specific guidelines/recommendations for the treatment of pediatric respiratory diseases with inhaled mucoactive drugs from an Indian perspective. Studies have shown that Indian children differ in the etiology and clinical presentation of certain pediatric respiratory conditions (e.g., CF, non-CF bronchiectasis) compared to Western populations (73–75). Furthermore, due to a lack of well-designed RCTs in this field of study in India, medical practitioners rely on data from the Western world. To the best of our knowledge, this is the first practical consensus document to assist pediatricians in clinical decision-making when selecting an appropriate mucoactive medication for the management of certain respiratory illnesses based on the most recent available information. Experts recommended inhaled mucoactive drugs (HS, mannitol, and dornase alfa) in children with CF. Inhaled HS was conditionally recommended for CF, acute bronchiolitis, and PCD. Experts agreed that inhaled HS before chest physiotherapy may be helpful in children with non-CF bronchiectasis, although more research into the pediatric population is required. The current state of evidence is inadequate to support the use of mucoactive drugs in asthma, wheezing, tracheomalacia tracheobronchomalacia, and EA with TEF. Currently, dornase alfa and mannitol dry powder are not available for use in India. Dornase alfa therapy is expensive, but the drug can be imported and is certainly useful in patients who can afford it. An alternative lower-cost therapy is inhaled HS, which has shown benefits in CF, non-CF bronchiectasis, PCD, and acute bronchiolitis. Currently, 3% and 7% HS concentrations of HS are accessible in India.

### 4.1. Strengths

The panelists were chosen from across India based on their level of clinical expertise, academic distinctions, and involvement in relevant clinical research. The expert committee was formed without any bias in terms of selection.

### 4.2. Limitation

The patient's voice was not included in the consensus process.

## 5. Conclusion

In this article, we have summarized clinical consensus statements/recommendations on the role of inhaled mucoactive drugs in pediatric respiratory disease conditions from an Indian perspective. Children with CF would benefit from the initiation of HS inhalation (as an adjunct to ACT) from the time of diagnosis. Clinical evidence supports the benefits of inhalation of rhDNase and mannitol dry powder in patients with CF; however, these drugs are currently not available in India. Experts suggested that inhaled salbutamol followed by HS in non-CF bronchiectasis and acute bronchiolitis may be beneficial. Inhaled salbutamol followed by inhaled HS can increase mucus clearance in children with PCD with underlying bronchiectasis and persistent weight cough similar to other non-CF bronchiectasis. Dornase alfa has been shown to reduce the length of stay and duration of ventilation in intubated and mechanically ventilated infants; however, more data are needed in this regard. The current state of evidence is inadequate to support the use of mucoactive drugs in asthma, wheezing, tracheomalacia, tracheobronchomalacia, and EA with TEF. Further, prospective RCTs are required to make a strong recommendation. Lastly, population-based studies are required to validate the effectiveness of inhaled mucoactive drugs in Indian children with specific respiratory conditions where mucus hypersecretion is a clinical challenge.

## References

[1] Linssen RSN Ma J Bem RA Rubin BK. Rational use of mucoactive medications to treat pediatric airway disease. Paediatr Respir Rev. (2020) 36:8–14. 10.1016/j.prrv.2020.06.007 32653467 PMC7297155

[2] Shen Y Huang S Kang J Lin J Lai K Sun Y Management of airway mucus hypersecretion in chronic airway inflammatory disease: Chinese expert consensus (English edition). Int J Chron Obstruct Pulmon Dis. (2018) 13:399–407. 10.2147/COPD.S144312 29430174 PMC5796802

[3] Knuuti J Wijns W Saraste A Capodanno D Barbato E Funck-Brentano C ESC Guidelines for the diagnosis and management of chronic coronary syndromes. Eur Heart J. (2019) 41(3):407–77. Published correction appears in Eur Heart J. 2020 November 21;41(44):4242. 10.1093/eurheartj/ehz425 31504439

[4] Schünemann H Broek J Guyatt G Oxman AD, Grade Working Group. GRADE handbook for grading quality of evidence and strength of recommendations. Available at: https://gdt.gradepro.org/app/handbook/handbook.html (Cited Jul 17 2023).

[5] Atkins D Best D Briss PA Eccles M Falck-Ytter Y Flottorp S Grading quality of evidence and strength of recommendations. Br Med J. (2004) 328(7454):1490. 10.1136/bmj.328.7454.1490 15205295 PMC428525

[6] Chen Q Shen Y Zheng J. A review of cystic fibrosis: basic and clinical aspects. Anim Models Exp Med. (2021) 4(3):220–32. 10.1002/ame2.12180 PMC8446696 34557648

[7] Wark P McDonald VM Smith S. Nebulised hypertonic saline for cystic fibrosis. Cochrane Database Syst Rev. (2023) 6(6):CD001506. 10.1002/14651858.CD001506.pub5 37319354 PMC10265937

[8] Ratjen F Davis SD Stanojevic S Kronmal RA Hinckley Stukovsky KD Jorgensen N Inhaled hypertonic saline in preschool children with cystic fibrosis (SHIP): a multicentre, randomised, double-blind, placebo-controlled trial. Lancet Respir Med. (2019) 7(9):802–9. 10.1016/S2213-2600(19)30187-0 31178421

[9] Tiddens HAWM Chen Y Andrinopoulou ER Davis SD Rosenfeld M Ratjen F The effect of inhaled hypertonic saline on lung structure in children aged 3-6 years with cystic fibrosis (SHIP-CT): a multicentre, randomised, double-blind, controlled trial. Lancet Respir Med. (2022) 10(7):669–78. 10.1016/S2213-2600(21)00546-4 35286860

[10] Elkins M Dentice R. Timing of hypertonic saline inhalation for cystic fibrosis. Cochrane Database Syst Rev. (2020) 2(2):CD008816. 10.1002/14651858.CD008816.pub4 32107770 PMC7046936

[11] Nevitt SJ Thornton J Murray CS Dwyer T. Inhaled mannitol for cystic fibrosis. Cochrane Database Syst Rev. (2020) 5(5):CD008649. 10.1002/14651858.CD008649.pub4 32358807 PMC7195814

[12] Sadr S Kiani M Rezaei M Khanbabaee G Tabatabaee SA Hosseini A. The efficacy of nebulized soluble mannitol and comparison with 5% hypertonic saline on pulmonary function of children with cystic fibrosis. J Compr Pediatr. (2019) 10(3):e85616. 10.5812/compreped.85616

[13] Yang C Montgomery M. Dornase alfa for cystic fibrosis. Cochrane Database Syst Rev. (2021) 3(3):CD001127. 10.1002/14651858.CD001127.pub5 33735508 PMC8094421

[14] Duijvestijn YC Brand PL. Systematic review of N-acetylcysteine in cystic fibrosis. Acta Paediatr. (1999) 88(1):38–41. 10.1111/j.1651-2227.1999.tb01265.x 10090545

[15] Donaldson SH Danielle Samulski T LaFave C Zeman K Wu J Trimble A A four-week trial of hypertonic saline in children with mild cystic fibrosis lung disease: effect on mucociliary clearance and clinical outcomes. J Cyst Fibros. (2020) 19(6):942–8. 10.1016/j.jcf.2020.07.009 32669217 PMC7736104

[16] Stahl M Wielpütz MO Ricklefs I Dopfer C Barth S Schlegtendal A Preventive inhalation of hypertonic saline in infants with cystic fibrosis (PRESIS). A randomized, double-blind, controlled study. Am J Respir Crit Care Med. (2019) 199(10):1238–48. 10.1164/rccm.201807-1203OC 30409023

[17] Elkins MR Robinson M Rose BR Harbour C Moriarty CP Marks GB A controlled trial of long-term inhaled hypertonic saline in patients with cystic fibrosis. N Engl J Med. (2006) 354(3):229–40. 10.1056/NEJMoa043900 16421364

[18] De Boeck K Haarman E Hull J Lands LC Moeller A Munck A Inhaled dry powder mannitol in children with cystic fibrosis: a randomised efficacy and safety trial. J Cyst Fibros. (2017) 16(3):380–7. 10.1016/j.jcf.2017.02.003 28258928

[19] Aitken ML Bellon G De Boeck K Flume PA Fox HG Geller DE Long-term inhaled dry powder mannitol in cystic fibrosis: an international randomized study. Am J Respir Crit Care Med. (2012) 185(6):645–52. 10.1164/rccm.201109-1666OC 22198974

[20] Fuchs HJ Borowitz DS Christiansen DH Morris EM Nash ML Ramsey BW Effect of aerosolized recombinant human DNase on exacerbations of respiratory symptoms and on pulmonary function in patients with cystic fibrosis. The pulmozyme study group. N Engl J Med. (1994) 331(10):637–42. 10.1056/NEJM199409083311003 7503821

[21] Quan JM Tiddens HA Sy JP McKenzie SG Montgomery MD Robinson PJ A two-year randomized, placebo-controlled trial of dornase alfa in young patients with cystic fibrosis with mild lung function abnormalities. J Pediatr. (2001) 139(6):813–20. 10.1067/mpd.2001.118570 11743506

[22] Serisier DJ Shute JK Hockey PM Higgins B Conway J Carroll MP. Inhaled heparin in cystic fibrosis. Eur Respir J. (2006) 27(2):354–8. 10.1183/09031936.06.00069005 16452592

[23] Máiz Carro L Martínez-García MA. Nebulized hypertonic saline in noncystic fibrosis bronchiectasis: a comprehensive review. Ther Adv Respir Dis. (2019) 13(13):1753466619866102. 10.1177/1753466619866102 31390940 PMC6688147

[24] Welsh EJ Evans DJ Fowler SJ Spencer S. Interventions for bronchiectasis: an overview of cochrane systematic reviews. Cochrane Database Syst Rev. (2015) 2015(7):CD010337. 10.1002/14651858.CD010337.pub2 26171905 PMC7086475

[25] Tarrant BJ Le Maitre C Romero L Steward R Button BM Thompson BR Mucoactive agents for chronic, non-cystic fibrosis lung disease: a systematic review and meta-analysis. Respirology. (2017) 22(6):1084–92. 10.1111/resp.13047 28397992

[26] https://www.brit-thoracic.org.uk/document-library/guidelines/bronchiectasis/bts-guideline-for-non-cf-bronchiectasis/.

[27] Anuradha KWDA Gunathilaka PKG Wickramasinghe VP. Effectiveness of hypertonic saline nebulization in airway clearance in children with non-cystic fibrosis bronchiectasis: a randomized control trial. Pediatr Pulmonol. (2021) 56(2):509–15. 10.1002/ppul.25206 33295693

[28] Kellett F Redfern J Niven RM. Evaluation of nebulised hypertonic saline (7%) as an adjunct to physiotherapy in patients with stable bronchiectasis. Respir Med. (2005) 99(1):27–31. 10.1016/j.rmed.2004.05.006 15672845

[29] Kellett F Robert NM. Nebulised 7% hypertonic saline improves lung function and quality of life in bronchiectasis. Respir Med. (2011) 105(12):1831–5. 10.1016/j.rmed.2011.07.019 22018993

[30] Nicolson CH Stirling RG Borg BM Button BM Wilson JW Holland AE. The long-term effect of inhaled hypertonic saline 6% in non-cystic fibrosis bronchiectasis. Respir Med. (2012) 106(5):661–7. 10.1016/j.rmed.2011.12.021 22349069

[31] Bilton D Daviskas E Anderson SD Kolbe J King G Stirling RG Phase 3 randomized study of the efficacy and safety of inhaled dry powder mannitol for the symptomatic treatment of non-cystic fibrosis bronchiectasis. Chest. (2013) 144(1):215–25. 10.1378/chest.12-1763 23429964

[32] Bilton D Tino G Barker AF Chambers DC De Soyza A Dupont LJ Inhaled mannitol for non-cystic fibrosis bronchiectasis: a randomised, controlled trial. Thorax. (2014) 69(12):1073–9. 10.1136/thoraxjnl-2014-205587 25246664

[33] Wills PJ Wodehouse T Corkery K Mallon K Wilson R Cole PJ. Short-term recombinant human DNase in bronchiectasis. Effect on clinical state and in vitro sputum transportability. Am J Respir Crit Care Med. (1996) 154(2 Pt 1):413–7. 10.1164/ajrccm.154.2.8756815 8756815

[34] O’Donnell AE Barker AF Ilowite JS Fick RB. Treatment of idiopathic bronchiectasis with aerosolized recombinant human DNase I. RhDNase study group. Chest. (1998) 113(5):1329–34. 10.1378/chest.113.5.1329 9596315

[35] Ater D Shai H Bar BE Fireman N Tasher D Dalal I Hypertonic saline and acute wheezing in preschool children. Pediatrics. (2012) 129(6):e1397–403. 10.1542/peds.2011-3376 22614767

[36] Kanjanapradap T Deerojanawong J Sritippayawan S Prapphal N. Does nebulized hypertonic saline shorten hospitalization in young children with acute viral wheezing? Pediatr Pulmonol. (2018) 53(2):138–44. 10.1002/ppul.23924 29266863

[37] Teper A Kofman C Alchundia Moreira J Köhler T García Bournissen F. Bronchodilator response to albuterol nebulized with hypertonic saline in asthmatic children. Pediatr Pulmonol. (2021) 56(12):3714–9. 10.1002/ppul.25653 34499820

[38] Barbato A Frischer T Kuehni CE Snijders D Azevedo I Baktai G Primary ciliary dyskinesia: a consensus statement on diagnostic and treatment approaches in children. Eur Respir J. (2009) 34(6):1264–76. 10.1183/09031936.00176608 19948909

[39] Kumar A Walker WT. Management of a child with primary ciliary dyskinesia. Oxf Med Case Rep. (2020) 2020(2):omz135. 10.1093/omcr/omz135 PMC7048077 32128223

[40] El-Abiad NM Clifton S Nasr SZ. Long-term use of nebulized human recombinant DNase1 in two siblings with primary ciliary dyskinesia. Respir Med. (2007) 101(10):2224–6. 10.1016/j.rmed.2007.05.014 17601719

[41] Paff T Daniels JM Weersink EJ Lutter R Vonk Noordegraaf A Haarman EG. A randomised controlled trial on the effect of inhaled hypertonic saline on quality of life in primary ciliary dyskinesia. Eur Respir J. (2017) 49(2):1601770. 10.1183/13993003.01770-2016 28232410

[42] Kaspy K Alarie N Vallee-Smedja S Adam J Shapiro DF. Initiation of nebulized hypertonic saline in infants with primary ciliary dyskinesia. Am J Respir Crit Care Med. (2020) et al;201:A7768. 10.1164/ajrccm-conference.2020.201.1_MeetingAbstracts.A7768.

[43] Stafanger G Garne S Howitz P Morkassel E Koch C. The clinical effect and the effect on the ciliary motility of oral N-acetylcysteine in patients with cystic fibrosis and primary ciliary dyskinesia. Eur Respir J. (1988) 1(2):161–7. 10.1183/09031936.93.01020161 3282911

[44] Anand R McAuley DF Blackwood B Yap C ONeill B Connolly B Mucoactive agents for acute respiratory failure in the critically ill: a systematic review and meta-analysis. Thorax. (2020) 75(8):623–31. 10.1136/thoraxjnl-2019-214355 32513777 PMC7402561

[45] Shein SL Gallagher JT Deakins KM Weinert DM. Prophylactic use of nebulized hypertonic saline in mechanically ventilated children: a randomized blinded pilot study. Respir Care. (2016) 61(5):586–92. 10.4187/respcare.04418 26732142

[46] Ezzeldin Z Mansi Y Gaber M Zakaria R Fawzy R Mohamed MA. Nebulized hypertonic saline to prevent ventilator associated pneumonia in premature infants, a randomized trial. J Matern Fetal Neonatal Med. (2018) 31(22):2947–52. 10.1080/14767058.2017.1359826 28738709

[47] Riethmueller J Borth-Bruhns T Kumpf M Vonthein R Wiskirchen J Stern M Recombinant human deoxyribonuclease shortens ventilation time in young, mechanically ventilated children. Pediatr Pulmonol. (2006) 41(1):61–6. Published correction appears in Pediatr Pulmonol. 2006;41(4):388. 10.1002/ppul.20298 16265663

[48] Youness HA Mathews K Elya MK Kinasewitz GT Keddissi JI. Dornase alpha compared to hypertonic saline for lung atelectasis in critically ill patients. J Aerosol Med Pulm Drug Deliv. (2012) 25(6):342–8. 10.1089/jamp.2011.0954 22413805

[49] Zitter JN Maldjian P Brimacombe M Fennelly KP. Inhaled dornase alfa (Pulmozyme) as a noninvasive treatment of atelectasis in mechanically ventilated patients. J Crit Care. (2013) 28(2):218.e1–e 7. 10.1016/j.jcrc.2012.09.015 23266402

[50] Masoompour SM Anushiravani A Tafaroj Norouz A. Evaluation of the effect of nebulized N-acetylcysteine on respiratory secretions in mechanically ventilated patients: randomized clinical trial. Iran J Med Sci. (2015) 40(4):309–15. 26170516 PMC4487455

[51] Wallis C Alexopoulou E Antón-Pacheco JL Bhatt JM Bush A Chang AB ERS Statement on tracheomalacia and bronchomalacia in children. Eur Respir J. (2019) 54(3):1900382. 10.1183/13993003.00382-2019 31320455

[52] Kamran A Jennings RW. Tracheomalacia and tracheobronchomalacia in pediatrics: an overview of evaluation, medical management, and surgical treatment. Front Pediatr. (2019) 7:512. 10.3389/fped.2019.00512 31921725 PMC6922019

[53] Goyal V Masters IB Chang AB. Interventions for primary (intrinsic) tracheomalacia in children. Cochrane Database Syst Rev. (2012) 10:CD005304. 10.1002/14651858.CD005304.pub3 23076914 PMC11613127

[54] Singh G Pandey A Shandilya G Gupta A Rawat JD Wakhlu A Evaluation of nebulized N-acetyl cysteine in outcome of esophageal atresia with tracheoesophegeal fistula. J Pediatr Surg. (2020) 55(12):2635–9. 10.1016/j.jpedsurg.2020.04.013 32467034

[55] Pandey A Gangopadhyay AN Sharma SP Kumar V. Esophageal atresia with tracheo-esophageal fistula: role of nebulized N-acetylcysteine in the outcome. J Indian Assoc Pediatr Surg. (2009) 14(4):232. 10.4103/0971-9261.59612 20419032 PMC2858892

[56] Boogaard R de Jongste JC Vaessen-Verberne AA Hop WC Merkus PJ. Recombinant human DNase in children with airway malacia and lower respiratory tract infection. Pediatr Pulmonol. (2009) 44(10):962–9. 10.1002/ppul.21073 19768804

[57] Porcaro F Valfré L Aufiero LR Dall’Oglio L De Angelis P Villani A Respiratory problems in children with esophageal atresia and tracheoesophageal fistula. Ital J Pediatr. (2017) 43(1):77. 10.1186/s13052-017-0396-2 28870218 PMC5584000

[58] Zhang L Mendoza-Sassi RA Wainwright C Aregbesola A Klassen TP. Nebulised hypertonic saline solution for acute bronchiolitis in infants. Cochrane Database Syst Rev. (2017) 12:CD006458. 10.1002/14651858.CD006458.pub4 29265171 PMC6485976

[59] Joshi K Parihar AS Singh J. Effect of nasal suction on reliving feeding difficulty in children affected with bronchiolitis. Int J Contemp Pediatr. (2020) 7(1):168–72. 10.18203/2349-3291.ijcp20195748

[60] Zhang L Mendoza-Sassi RA Wainwright CE Klassen TP. Nebulised hypertonic saline solution for acute bronchiolitis in infants. Cochrane Database Syst Rev. (2023) 4(4):CD006458. 10.1002/14651858.CD006458.pub5 37014057 PMC10072872

[61] Enriquez A Chu IW Mellis C Lin WY. Nebulised deoxyribonuclease for viral bronchiolitis in children younger than 24 months. Cochrane Database Syst Rev. (2012) 11(11):CD008395. 10.1002/14651858.CD008395.pub2 23152257 PMC7388903

[62] Naz F Raza AB Ijaz I Kazi MY. Effectiveness of nebulized N-acetylcysteine solution in children with acute bronchiolitis. J Coll Physicians Surg Pak. (2014) 24(6):408–11. 24953914

[63] Zhang L Gunther CB Franco OS Klassen TP. Impact of hypertonic saline on hospitalization rate in infants with acute bronchiolitis: a meta-analysis. Pediatr Pulmonol. (2018) 53(8):1089–95. 10.1002/ppul.24066 29893029

[64] Islam MS Mollah MAH Khanam R Chowdhury AS Rahman MM Al Baqui SAA Comparative efficacy of nebulized 7% hypertonic saline versus 0.9% normal saline with salbutamol in children with acute bronchiolitis. Bangladesh J Child Health. (2019) 43(2):80–4. 10.3329/bjch.v43i2.42550

[65] Yu JF Zhang Y Liu ZB Wang J Bai LP. 3% Nebulized hypertonic saline versus normal saline for infants with acute bronchiolitis: a systematic review and meta-analysis of randomized controlled trials. Medicine (Baltim). (2022) 101(43):e31270. 10.1097/MD.0000000000031270 PMC10662888 36316926

[66] Gibson PG Grootendor DC Henry RL Pin I Rytila PH Wark P Sputum induction in children. Eur Respir J Suppl. (2002) 37:44s–6s. 10.1183/09031936.02.00004402 12361363

[67] Suri R Marshall LJ Wallis C Metcalfe C Shute JK Bush A. Safety and use of sputum induction in children with cystic fibrosis. Pediatr Pulmonol. (2003) 35(4):309–13. 10.1002/ppul.10226 12629630

[68] Ferreira ACM Marson FAL Cohen MA Bertuzzo CS Levy CE Ribeiro AF Hypertonic saline as a useful tool for sputum induction and pathogen detection in cystic fibrosis. Lung. (2017) 195(4):431–9. 10.1007/s00408-017-0008-3 28455785

[69] Ronchetti K Tame JD Paisey C Thia LP Doull I Howe R The CF-sputum induction trial (CF-SpIT) to assess lower airway bacterial sampling in young children with cystic fibrosis: a prospective internally controlled interventional trial. Lancet Respir Med. (2018) 6(6):461–71. 10.1016/S2213-2600(18)30171-1 29778403 PMC5971213

[70] Popov TA Pizzichini MM Pizzichini E Kolendowicz R Punthakee Z Dolovich J Some technical factors influencing the induction of sputum for cell analysis. Eur Respir J. (1995) 8(4):559–65. 10.1183/09031936.95.08040559 7664854

[71] Seong GM Lee J Lee JH Kim JH Kim M. Usefulness of sputum induction with hypertonic saline in a real clinical practice for bacteriological yields of active pulmonary tuberculosis. Tuberc Respir Dis (Seoul). (2014) 76(4):163–8. 10.4046/trd.2014.76.4.163 24851129 PMC4021263

[72] Brown M Varia H Bassett P Davidson RN Wall R Pasvol G. Prospective study of sputum induction, gastric washing, and bronchoalveolar lavage for the diagnosis of pulmonary tuberculosis in patients who are unable to expectorate. Clin Infect Dis. (2007) 44(11):1415–20. 10.1086/516782 17479935

[73] Prasad R Sharma H Kaur G. Molecular basis of cystic fibrosis disease: an Indian perspective. Indian J Clin Biochem. (2010) 25(4):335–41. 10.1007/s12291-010-0091-1 21966101 PMC2994562

[74] Kumar A Lodha R Kumar P Kabra SK. Non-cystic fibrosis bronchiectasis in children: clinical profile, etiology and outcome. Indian Pediatr. (2015) 52(1):35–7. 10.1007/s13312-015-0563-8 25638182

[75] Chandrasekaran R Mac Aogáin M Chalmers JD Elborn SJ Chotirmall SH. Geographic variation in the aetiology, epidemiology and microbiology of bronchiectasis. BMC Pulm Med. (2018) 18(1):83. 10.1186/s12890-018-0638-0 29788932 PMC5964678

