# Supplementary material for: Expert group recommendation on inhaled mucoactive drugs in pediatric respiratory diseases: an Indian perspective
Source: Front Pediatr. 2023 Dec 4;11:1322360. doi: 10.3389/fped.2023.1322360 (PMC10725989; doi:10.3389/fped.2023.1322360)
Supplement: Supplementary file 1 [file Table1.docx]

Supplementary Material

Expert Group Recommendation on Inhaled Mucoactive Drugs in Pediatric Respiratory Diseases: An Indian Perspective

Meenu Singh^1*^, Sneha Varkki^2^, Ilin Kinimi^3^, Rashmi R Das^4^, Jagdish Goyal^5^, Mushtaq Bhat^6^, Rajeshwar Dayal^7^, Pawan Kalyan^8^, Jitender Gairolla^9^, Indu Khosla^10^

*** Correspondence:** Meenu Singh: meenusingh4@gmail.com

# Supplementary Data

**Table S1: Demographic and Professional Characteristics of Expert Panel**

| **Panelist no.** | **Gender** | **Age (years)** | **Zone** | **Geographic location** | **Clinical experience**  **(years)** | **Specialty** | **Type of practice** | **Patient load/day** |
| --- | --- | --- | --- | --- | --- | --- | --- | --- |
| 1 | Female | 64 | North | Rishikesh, India | 37 | Pediatrics | Institutional clinical practice | 70–100 |
| 2 | Female | 49 | South | Bangalore, India | 18 | Pediatrics | Private clinical practice | 60–70 |
| 3 | Male | 47 | West | Jodhpur, India | 20 | Pediatrics | Institutional clinical practice | 100–150 |
| 4 | Male | 45 | East | Bhubaneswar, India | 15 | Pediatrics | Institutional clinical practice | 100 |
| 5 | Male | 42 | South | Vijayawada, India | 11 | Pediatrics | Private clinical practice | 70–80 |
| 6 | Male | 55 | North | Srinagar, India | 25 | Pediatrics | Institutional clinical practice | 100 |
| 7 | Male | 39 | North | Rishikesh, India | 11 | Microbiologist | Research Scientist |  |
| 8 | Female | 55 | South | Vellore, India | 25 | Pediatrics | Institutional clinical practice, tertiary care | 100–120 |
| 9 | Male | 65 | North | Agra, India | 38 | Pediatrics | Institutional clinical practice | 150 |
| 10 | Female | 60 | West | Mumbai, India | 35 | Pediatrics | Private and corporate clinical practice | 70–100 |

**Table S2: Questionnaire**

| **Major domains** | **Questions** |
| --- | --- |
| Cystic fibrosis | 1. What is the role of nebulized mucoactive drugs in the treatment of cystic fibrosis? 2. Which nebulized mucoactive drugs should be used in pediatric cystic fibrosis?   What is the recommended dose and duration of therapy? (Hypertonic saline, N-acetyl cysteine, dornase alfa, mannitol, dextran, heparin, gelsolin, any other) |
| Noncystic fibrosis bronchiectasis | 1. What is the role of nebulized mucoactive drugs in the treatment of non-cystic fibrosis bronchiectasis? 2. Which nebulized mucoactive drugs should be used in non-cystic fibrosis bronchiectasis?   What is the recommended dose and duration of therapy? (Hypertonic saline, N-acetyl cysteine, any other) |
| Asthma and acute wheezing | 1. Based on your clinical experience, in which patient profiles (phenotypes) of asthma, is nebulized mucoactive treatment warranted? 2. Is there any role of nebulized mucoactive drugs in management of acute asthma and acute wheezing? If yes, which nebulized mucoactive drugs should be used?   What is the recommended dosage regimen and duration of therapy? (Hypertonic saline, N-acetyl cysteine, any other) |
| Primary ciliary dyskinesia | 1. What is the role of nebulized mucoactive drugs in children with primary ciliary dyskinesia?   Which nebulized mucoactive drugs should be used?  What is the recommended dose and duration of therapy? (Hypertonic saline, N-acetyl cysteine, any other) |
| - Critically ill on mechanical ventilator support - Tracheomalacia tracheobronchomalacia - Esophageal atresia/tracheoesophageal fistula | 1. What is the role of nebulized mucoactive drugs in children with the following conditions: (i) tracheomalacia; (ii) tracheobronchomalacia; and (iii) tracheoesophageal fistula/atresia?   Which nebulized mucoactive drugs should be used?  What is the recommended dose and duration of therapy? (Hypertonic saline, N-acetyl cysteine, any other)   1. What is the role of nebulized mucoactive drugs in children with acute respiratory failure on mechanical ventilator support?   Which nebulized mucoactive drug should be used?  What is the recommended dose and duration of therapy? (Hypertonic saline, N-acetyl cysteine, any other) |
| Acute bronchiolitis | 1. What is the role of nebulized mucoactive drugs in the treatment of bronchiolitis? 2. Which nebulized mucoactive drugs should be used in pediatric acute bronchiolitis?   What is the recommended dosage regimen and duration of therapy? (Hypertonic saline, N-acetyl cysteine, any other) |
| Sputum induction | 1. Do you think that nebulized mucoactive drugs can serve as a useful tool in the diagnosis of pediatric respiratory diseases where sputum induction is warranted?   Which nebulized mucoactive drugs do you recommend for sputum induction? What is the recommended dose and protocol for sputum induction? |
